# Persistent Left Superior Vena Cava and Absent Right Superior Vena Cava with Left Subclavian Vein Stenosis: Technical Challenges with Pacemaker Implantation

**DOI:** 10.1155/2019/7271591

**Published:** 2019-07-29

**Authors:** Khalil Kanjwal, Michael Soos, Daniel Gonzalez-Morales, Ibrahim Shah, Mohan Madala, Majid Mughal, Muhammad Awais Kang

**Affiliations:** McLaren Greater Lansing Hospital, Lansing, MI, USA

## Abstract

We present a challenging case of a 75-year-old female with a history of paroxysmal atrial fibrillation (PAF) and symptomatic sick sinus syndrome (SSS) who presented for a dual chamber pacemaker implantation and was found to have persistent left superior vena cava and absent right superior vena cava with stenosis of the left subclavian vein. In this report, we discuss the implant technique in this group of patients.

## 1. Introduction

Persistent left superior vena cava (PLSVC) along with absent right SVC is a rare occurrence. It has been reported in 0.1% of the general population [1–3]. There have been only less than 15 cases reported in the literature. Pacemaker implantation can be very challenging in such patients, and there has not been much reported on various techniques of implanting pacemaker in such patients. To our knowledge, this is a second report of pacemaker implantation in a patient with persistent left SVC and absent right SVC. In addition, our patient also had left subclavian vein stenosis and thus complicating the implant procedure further.

## 2. Case

A 75-year-old female with symptomatic paroxysmal atrial fibrillation (PAF), statuspost atrial fibrillation ablation, and prior history of breast cancer, statuspost bilateral mastectomy and left lymphadenectomy, presented with diziness and near syncope and was found to have sinus bradycardia. Given her symptomatic sick sinus syndrome (SSS), she was planned for a dual chamber pacemaker implantation.

**Figure 1:**
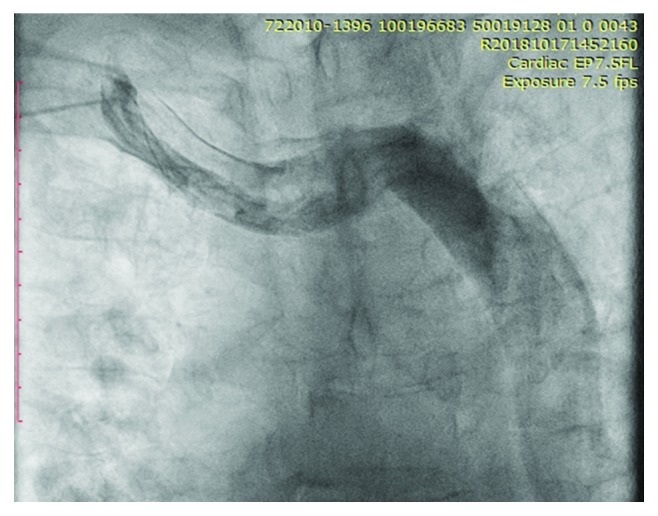


**Figure 2:**
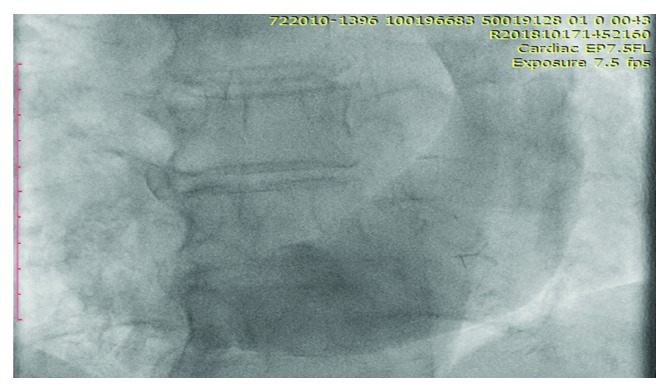


The placement of a dual chamber pacemaker insertion was attempted from the right side as the patient previously underwent left-sided lymphadenectomy. The right subclavian vein access was obtained and cannulated under fluoroscopy. The right ventricular (RV) lead was advanced attempting to access the right superior vena cava (SVC). While attempting to advance the lead, resistance was encountered and the lead was withdrawn. A venogram was performed revealing the absence of a right SVC with the presence of a persistent left superior vena cava draining into a massively dilated coronary sinus (Figures 1 and 2).

The lead could not be advanced into the right ventricle, and as a result, the procedure was aborted and cardiothoracic surgery was consulted for possible epicardial lead placement.

After discussing the potential need for cardiothoracic surgery with the patient, she considered another attempt at transvenous placement of a dual chamber pacemaker with us. After repeated attempts, left subclavian vein access could not be obtained. The venogram revealed stenosis of the left subclavian vein. The decision was made to make an additional attempt from the right subclavian vein utilizing the previous pocket. After successful cannulation of the right subclavian vein, an active fixation lead was advanced through the subclavian vein into the PLSVC through the coronary sinus and then through the tricuspid valve into the right ventricular septum. Following successful right ventricular lead placement, an active fixation bipolar pacing lead was advanced into the right atrium using a straight stylet and secured to the right atrial wall.

Stimulation and sensing thresholds were determined to be satisfactory in both leads. A St. Jude Medical generator was connected to the leads, and the pocket was closed.

## 3. Discussion

PLSVC is a rare congenital anomaly found in approximately 0.3-0.5% of the general population [1–3], and its occurrence along with absent right-sided SVC is reported in 0.1% of the general population. It results from the failure of the vessel to involute during embryologic development.

**Figure 3:**
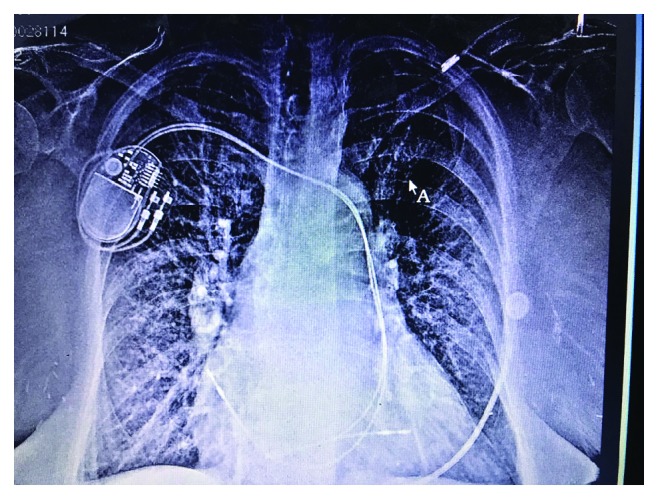


This anomaly usually exists with the presence of a right superior vena cava (SVC) and is usually found incidentally during cardiac device implantation. In patient with PLSVC, the cardiac devices are implanted from the right subclavian approach as the lead manipulation and deployment can be challenging from the left side. Absent right SVC with PLSVC occurs extremely rarely in less than 0.1% of the general population [1, 2]. In this report, we outline the technical approach to the placement of dual chamber pacemaker leads in a patient with solitary PLSVC. Our case was further complicated by subclavian stenosis on the left side. The right subclavian access was obtained using a micropuncture needle under fluoroscopy. The venography demonstrated the absence of right-sided SVC, and the wire could easily pass over the left SVC. The sheath was advanced over the wire, and a 65 cm active fixation lead could be easily advanced through the right subclavian into the left SVC and the coronary sinus and through the coronary sinus os into the right atrium in the right anterior oblique (RAO) fluoroscopic view. As the lead came out of the coronary sinus, it is directed in a direction opposite to that of the RV inlet, thus making the implantation and manipulation of the RV lead challenging. The most critical part of the procedure is crossing the tricuspid valve, and inability to do that will result in failure of the lead placement. In our patient, when the lead came out of the coronary sinus, it was further advanced without the stylet towards the right atrial wall. The forward movement against the RA free wall formed a loop, and subsequently, the part of the lead was prolapsed into the right ventricle. The stylet with a spiral curve made at the end of the stylet was introduced and advanced, and when it reached the coronary sinus os, the pull and push maneuver on the stylet and the lead was performed. The lead tip crossed the RV inlet and was further advanced into the RV towards RV septum. The septal location was confirmed in the left anterior oblique (LAO) fluoroscopic position, and subsequently, the helix was deployed (Figure 3).

There are few technical considerations the implanting physicians need to be aware of while implanting cardiac devices in patients with PLSVC and absent right SVC.

The course of the right subclavian to PLSVC is long. A longer sheath may be considered while navigating the lead through anomalous course of the vein. In addition, a longer lead (65 cm) would be a preferred length lead given the long course of right subclavian, PLSVC, and coronary sinus. The prolapse technique with a spiral curve of the stylet should be considered during such implants.

Other potential alternatives may be included using a coronary sinus deliver system. A 0.35^″^ wire may be advanced through the subclavian/left SVC venous system through coronary sinus os and through the RV inlet into the right ventricle followed by advancing the delivery system over the wire into the right ventricle.

The right atrial lead does not pose a technical challenge in such patients. When it comes out of the CS OS, it is directed towards the right free wall; however, it could be challenging sometimes to direct the lead into the right atrial appendage. A clockwise torque with a J-shaped stylet may help in such situation.

To the best of our knowledge, there has been only one report of pacemaker implantation in a patient with persistent left SVC with absent right SVC from the right subclavian vein [4]. The use of a fixed-shaped sheath and a lumen less bipolar pacing lead from Medtronic has been recently reported for implantation of a pacemaker from the left brachiocephalic system through the left persistent SVC [5].

## 4. Conclusion

Venous anomalies can be encountered during cardiac device implantations. Preprocedure venography may help identify such anomalies, and the physicians can plan on the techniques and need for special equipment ahead of time to have a better patient outcome.

## Additional Points

*Teaching Points*. (1) Preimplant venograms provide crucial information about venous anatomy. (2) Implanting physicians should be aware of venous anomalies encountered during device implants. (3) A clear concept of venous anomalies and trouble shooting skills may improve patient outcomes.
